# NS1 codon usage adaptation to humans in pandemic Zika virus

**DOI:** 10.1590/0074-02760170385

**Published:** 2018-05-10

**Authors:** Caio César de Melo Freire, Giuseppe Palmisano, Carla T Braconi, Fernanda R Cugola, Fabiele B Russo, Patricia CB Beltrão-Braga, Atila Iamarino, Daniel Ferreira de Lima, Amadou Alpha Sall, Livia Rosa-Fernandes, Martin R Larsen, Paolo Marinho de Andrade Zanotto

**Affiliations:** 1 Universidade Federal de São Carlos, Departamento de Genética e Evolução, São Carlos, SP, Brasil; 2 Universidade de São Paulo, Instituto de Ciências Biomédicas, Departamento de Parasitologia, São Paulo, SP, Brasil; 3 Universidade de São Paulo, Instituto de Ciências Biomédicas, Departamento de Microbiologia, Laboratório de Evolução Molecular e Bioinformática, São Paulo, SP, Brasil; 4 Universidade de São Paulo, Laboratório de Células-Tronco, Departamento de Cirurgia, São Paulo, SP, Brasil; 5 Universidade de São Paulo, Escola de Artes, Ciências e Humanidades, Departamento de Obstetrícia, São Paulo, SP, Brasil; 6 Institute Pasteur of Dakar, Dakar, Senegal; 7 University of Southern Denmark, Department of Biochemistry and Molecular Biology, Odense, Denmark

**Keywords:** Zika virus, codon usage biases, proteomics, NS1 protein

## Abstract

**BACKGROUND:**

Zika virus (ZIKV) was recognised as a zoonotic pathogen in Africa and southeastern Asia. Human infections were infrequently reported until 2007, when the first known epidemic occurred in Micronesia. After 2013, the Asian lineage of ZIKV spread along the Pacific Islands and Americas, causing severe outbreaks with millions of human infections. The recent human infections of ZIKV were also associated with severe complications, such as an increase in cases of Guillain-Barre syndrome and the emergence of congenital Zika syndrome.

**OBJECTIVES:**

To better understand the recent and rapid expansion of ZIKV, as well as the presentation of novel complications, we compared the genetic differences between the African sylvatic lineage and the Asian epidemic lineage that caused the recent massive outbreaks.

**FINDINGS:**

The epidemic lineages have significant codon adaptation in NS1 gene to translate these proteins in human and *Aedes aegypti* mosquito cells compared to the African zoonotic lineage. Accordingly, a Brazilian epidemic isolate (Z^BR^) produced more NS1 protein than the MR766 African lineage (Z^AF^) did, as indicated by proteomic data from infections of neuron progenitor cells-derived neurospheres. Although Z^BR^ replicated more efficiently in these cells, the differences observed in the stoichiometry of ZIKV proteins were not exclusively explained by the differences in viral replication between the lineages.

**MAIN CONCLUSIONS:**

Our findings suggest that natural, silent translational selection in the second half of 20th century could have improved the fitness of Asian ZIKV lineage in human and mosquito cells.

Zika virus (ZIKV) was known as a zoonotic pathogen with sporadic human infections in Africa and later in southeastern Asia (Hayes 2009), until the spread of its Asian lineage along the Pacific Islands and Americas, vectored mainly by *Aedes aegypti* (Musso and Cao-Lormeau 2015) and marginally by alternative routes of transmission-body fluids, sexual intercourse and perinatal infections. In early 2015, it was detected by molecular techniques in Brazil [Supplementary data (Fig. 1)], where it caused a severe epidemic and produced novel symptoms and post-infection complications (Possas et al. 2017).

Zika fever (ZF) symptoms include persisting arthralgia, headaches and mild fever. The recent ZIKV outbreaks were also associated with a 20-fold increase in cases of Guillain-Barre syndrome (GBS) in French Polynesia, where 98% of the GBS patients showed anti-ZIKV antibodies (Cao-Lormeau et al. 2016). An increase in the number of GBS cases was also observed in the Brazilian state Bahia, where ZIKV transmission was simultaneous with Dengue virus (DENV) and Chikungunya virus (CHIV). The incidence of ZF-like syndrome reached 328 cases per 100,000 inhabitants from May to August 2015 (Possas et al. 2017). Worryingly, ZIKV was also associated with the abrupt increase of newborns with birth defects (Possas et al. 2017). To date, congenital ZIKV syndrome (CZS) has been causally associated with the Asian lineage of the virus (Cugola et al. 2016) but further studies with other ZIKV lineages are needed.

Viral spillover frequently depends on host adaptation through evolution. One crucial evolutionary mechanism and an indicator of viral adaptation to the host are changes in nucleotide composition (Longdon et al. 2014). Codon usage adaptation after a host shift event could be required to fine-tune the interactions between a virus and a new host (Bahir et al. 2009, Longdon et al. 2014). These changes are not random. Rather, they occur preferentially at interaction sites, such as in viral glycoproteins. Whether these evolutionary changes were important for the ZIKV Asian lineage that has spread from Asia to the Americas remains an open question. To better understand aspects of its recent circulation, we investigated evolutionary changes in codon usage of ZIKV lineages, employing sequence analyses and comparative proteomics between African and epidemic Asian lineages.

## MATERIALS AND METHODS

*Sequence datasets* - We investigated 42 representative, available complete genome sequences of ZIKV from GenBank (www.ncbi.nlm.nih.gov/genbank/) that had information of year and country of isolation (alignment available from: https://github.com/CaioFreire/CUB). First, we aligned the coding sequences with MACSE program v0.9 (https://bioweb.supagro.inra.fr/macse/) and curated it with AliView v1.171 (http://ormbunkar.se/aliview/). Since we previously found evidence for recombination in ZIKV from Africa (Faye et al. 2014) and these events could cause potential errors in phylogenetic inferences, we screened for recombination in genomic sequences with the RDP program v4.36 (http://web.cbio.uct.ac.za/~darren/rdp.html). Recombinants (Accession numbers KF383116, KF383117, KF383118 and KU866423) that were identified by more than three independent methods with p-value < 0.05, using Bonferroni correction, were removed of selection and phylodynamic analyses.

*Codon preferences analyses* - We employed the relative synonymous codon usage method with the R-package Seqin{R} v3.13 (http://seqinr.r-forge.r-project.org/) to estimate the codon preferences for each polyprotein gene sequence. In addition, we employed a principal component analysis (PCA) to assess patterns among relative synonymous codon usage (RSCU) values for all the codons, among viral lineages. We identified the most informative codons, which were informative to discriminate among Asian and African lineages, with a biplot graph for the PCA values with the R-package ggbiplot v0.55 (https://github.com/vqv/ggbiplot), using a group probability of 0.95. The different codon preferences between ZIKV lineages were independently confirmed by high support values (> 80%) obtained from hierarchical clustering analysis, using the R-package Pvclust v1.32 (http://stat.sys.i.kyoto-u.ac.jp/prog/pvclust/). We also investigated the differences in codon preferences between ZIKV lineages, employing a PCA in codon counts, which were calculated with Seqin{R}.

*Codon usage biases* - We calculated the effective number of codons (ENC) with Emboss v6.60 (http://emboss.sourceforge.net/) and the proportion of guanine-cytosine content in the third base of the codons (GC_3_), using the Seqin{R} program to evaluate the codon usage bias (CUB). The theoretical curve of ENC x GC_3_ on the genetic drift was estimated with a Perl script to calculate expected ENC and GC_3_ values (available from: https://github.com/CaioFreire/CUB). We inspected the amino acid content of each gene with Seqin{R} and compared the averages of each amino acid content between the ZIKV lineages with Fisher exact tests.

*Codon adaptation of ZIKV genes to humans and Ae. aegypti mosquitoes* - In our analysis, CAI is a measure of synonymous CUB based on the codon preference of a viral strain and a codon usage table for a given host. To investigate if the codon usage of ZIKV lineages was similar to the hosts in urban settings, regarding humans and Ae. aegypti, we calculated the codon adaptation index (CAI) for each gene from each ZIKV lineage. Instead of random sampling of human genes to calculate the codon usage table (Cristina et al. 2016) and because the most pronounced biases are in highly expressed genes (Bahir et al. 2009), we used Emboss to calculate a codon usage table for humans (available from: https://github.com/CaioFreire/CUB) based on 3803 genes identified as housekeeping (Eisenberg and Levanon 2013), which are strongly and uniformly expressed in 16 humans tissues. Moreover, we calculated CAI for Ae. aegypti using the table available in Codon Usage Database (http://www.kazusa.or.jp/codon/). Importantly, the CAI values obtained with our table based on housekeeping genes were very similar to those found with the table from Codon Usage Database with generic human genes. The CAI values for each sequence from ZIKV genes were calculated with CAIcal program (http://genomes.urv.es/CAIcal/). We assessed the confidence of CAI estimates by the calculation of expected CAI values for 1000 random sequences with similar GC-content and the same length of the query sequence. We compared the CAI values between Asian and African lineages with Wilcoxon rank sum test. The sliding window analyses were managed with a Perl script (available from: https://github.com/CaioFreire/CUB) that used the CAI program from Emboss package v6.6.0 to calculate the CAI values for each window with 25 codons.

*Viral culture, amplification and titration* - We used a lyophilised ZIKV (Z^BR^ - BeH815744) isolated from a febrile case in the state of Paraiba in the northeast of Brazil isolated in the Evandro Chagas Institute (IEC) in Belém do Pará, Brazil. This virus was reconstituted in 0.5 mL of sterile diethylpyrocarbonate (DEPC) water. The African-lineage (Z^AF^ - MR-766), which is a reference strain isolated in Uganda in 1947, was provided by the Institute Pasteur in Dakar, Senegal. Both strains were cultured and amplified in cells of *Aedes albopictus* (C6/36 cells). The C6/36 cells were maintained using Leibovitz’s L-15 medium and supplemented with 10% foetal bovine serum (FBS) (Gibco), 1% non-essential amino acids (Gibco), 1% sodium pyruvate (Gibco), 1% penicillin/streptomycin (Gibco), 0.05% of amphotericin B (Gibco). The C6/36 cells were kept at 27ºC in the absence of CO2 and tested negative for mycoplasma contamination. After reaching 70% confluent monolayer, 50 µL of Z^BR^ was inoculated into C6/36 cells and left adsorb into cells for 1 h, with gentle shaking every 10 min. The cultures were then incubated under the same adsorption conditions. We used the titrated third passage for experimental inoculation (Cugola et al. 2016). The virus titration (in PFU mL-1) of each C6/36 subculture was obtained by plaque assay in porcine kidney epithelial (PS) cells, according to (Cugola et al. 2016). For Z^BR^, the first C6/36 subculture (T1) had a viral titer of 6 × 10^8^. The T2 and T3 subcultures had titers of 7.5 × 10^6^ and 4 × 10^12^, respectively. For Z^AF^, the first C6/36 subculture (T1) had a titer of 1.0 × 10^6^ and 7.5 × 10^12^ in T2. All the subculture aliquots were stored in cryovials and maintained in liquid nitrogen. For the infections before proteomics we also used third passage inoculum of Z^BR^ and a second passage obtained in our lab for Z^AF^.

*Neurospheres culture* - We used a human induced pluripotent stem cell (iPSC) clone that was previously characterised in the Beltrão-Braga and Muotri laboratories (Cugola et al. 2016). This cell line tested negative for mycoplasma contamination. Briefly, high passages of iPSC colonies on feeder-free plates were maintained for five days with mTSeR media (Stem Cell Technologies). On the fifth day, the medium was changed to N2 media (DMEM/F12 medium supplemented with 1X N2 supplement (Invitrogen) and the dual SMAD inhibitors, 1 μM dorsomorphin (Tocris) and 1 μM SB431542 (Stemgent), for 48 h. Further, the colonies were detached from the plate and cultured in suspension as embryoid bodies (EBs) for five days at 90 r.p.m. in N2 media with the dual SMAD inhibitors. The EBs were plated on matrigel-coated plates with NBF media composed of the following: DMEM/F12 media supplemented with 0.5X N2, 0.5X B27 supplement (Gibco), 20 ng mL-1 of fibroblast growth factor 2 (FGF2) and 1% penicillin/streptomycin. The emerged rosettes containing the NPCs were manually picked, dissociated and plated in a double-coated plate with poly-ornithine (10 μg mL-1, Sigma-Aldrich) and laminin (2.5 μg mL-1, Gibco). The NPC population was expanded using NBF media. To produce neurospheres, NPC were scrapped from the plates and submitted to continuous shaking at 90 rpm in NBF medium. All experiments were performed with the approval of the Institute of Biomedical Sciences Ethics Committee (protocol number 1001).

*In vitro infection* - Neurospheres were infected with Z^BR^ -BeH815744, Z^AF^- MR-766 and mock (culture supernatant from uninfected C6/36 cells). NPCs were seeded in 100mm plates and after 24 h viral samples were diluted to the desired multiplicity of of infection (MOI) of 10 and added to the cells. For viral adsorption, cells in monolayer were incubated for 1 h at 4ºC with gentle agitation every 10 min. Next, the inoculum was removed and cells were washed once with PBS (USB Corporation). Culture medium was added to each well, and cells were incubated at 37ºC and 5% CO2 in constant shaking for the duration of the experiment. For mock controls, the same volume of supernatant was added to each experiment, and the same procedures were followed.

*Experimental setup, protein extraction and tryptic digestion* - Cells infected with the African (Z^AF^- MR-766) strain, the Brazilian (Z^BR^ -BeH815744) viral strain and a mixture of the two viruses were labeled with the neutron encoded TMT 10-Plex, as described below. Three biological replicates for each condition were analysed. In particular, cells infected with the African strain were labeled with 126, 127N and 127C. Cells infected with the Brazilian strain were labeled with 128C, 128N and 129C. Cells infected with a mixture of the two viruses were labelled with 129N, 130C and 130N. Further details on the labeling protocol are reported below. Two million cells per condition were lysed in 500 uL of HENS buffer containing 100 mM HEPES (pH 7.8), 1 mM EDTA, 0.1 mM Neocuproine, and 1% sodium docecyl sulfate (SDS) with the addition of protease and phosphatase inhibitors. In order to block free cysteines, N-ethyl maleimide was added (40mM). The cell lysate was incubated for 20 min on ice before the addition of four volumes of ice-cold acetone. The mixture was incubated overnight at -20ºC. Protein were centrifuged at 14000 g for 10 min. Protein pellet was re-suspended in 8 M urea in 100 mM TEAB and proteins quantified using Qubit (Invitrogen). Disulfide bonds were reduced with 10 mM dithiothreitol DTT and alkylated with 40 mM IAA. Proteins were digested with trypsin at 1:50 enzyme: substrate ratio. Tryptic peptides were desalted with Oasis HLB (Waters) before quantification using Qubit.

*Peptide labelling with neutron encoded TMT 10-Plex* - One hundred micrograms of peptides were labeled with TMT 10-plex (Thermo Fisher Scientific) according to manufacturer instructions. Briefly, peptides were resuspended in 20 mM TEAB and the TMT 10-plex reagent, dissolved in acetonitrile, was added. After 1 h, the reaction was blocked with hydroxylamine, incubated for 15 min before the different conditions were combined in a 1:1:1:1:1:1:1:1:1 proportion. The combined peptide mixture was desalted using reversed-phase microcolumns before hydrophilic interaction chromatography (HILIC) fractionation, as previously described (Palmisano et al. 2010).

*Nanoscale liquid chromatography coupled to tandem mass spectrometry analysis* - Each HILIC fraction was dried in vacuum and resuspended in 0.1% formic acid in water. The sample was injected into an Easy-nanoLC 1000 (Thermo Fisher Scientific) coupled to a Q Exactive (Thermo Fisher Scientific). Peptides were initially loaded onto a precolumn 3.5 cm x 100 um (i.d.) and separated on custom-made 18 cm × 75 μm (i.d.) reversed-phase columns (Reprosil). Gradient elution was performed from 0% acetonitrile to 28% acetonitrile in 0.1% formic acid over 52 min up to 45% acetonitrile to 57 min. The total length of the run was set to 70 min. The instrument was operated with a data dependent top 15 method. Mass spectrometry (MS) spectra were acquired using a Q Exactive instrument 120.000 resolution in a range of 400-1600 m/z and HCD scans at 30.000 resolution (at m/z 200). The MS ion target was 3e6. MS2 ion target was set to 1e5 with a maximum injection time of 150 ms and isolation window of 1.2 m/z. The raw files are available from the authors upon request.

*Database search* - Raw data were searched with MaxQuant (Cox and Mann 2008) and the database search engine Andromeda. Uniprot database contained human proteins, Zika virus proteins (PE_243 and MR_766) and common contaminants. MS accuracy was set to 4.5 ppm and MS/MS accuracy to 20 ppm. Trypsin was set as cleavage enzyme with two missed cleavages. Carbamidomethylation of cysteine, N-ethyl maleimide of cysteine and oxidation of methionine were set as variable modifications. All identifications were filtered in order to achieve a FDR of less than 1%. Protein/peptide quantification was performed using the reporter ion MS2 module with the TMT 10-plex, embedded in the MaxQuant software. Protein quantification was performed using razor and unique peptides. Reporter ion intensities were given and assembled into the protein intensity. Each reporter ion intensity was corrected and normalised for the total intensity, so any bias due to sample loading was corrected. After excluding peptides identified as potential contaminants or in reverse database, peptides belonging to the ZIKV were manually filtered and analysed. The relative protein expression was assembled using the top 3 most intense peptides for each protein. Some proteins such as NS2A, NS2B and NS3 were identified with a single peptide. The ratio of proteins identified and quantified in neurospheres infected with Z^BR^ and Z^AF^ was reported. In order to check for sample variability between the different biological replicates, the log2(Intensity) for each protein was compared and reported as a scatter plot. The three biological replicates of neurospheres infected with Z^AF^ (A1, A2 and A3) and three biological replicates of neurospheres infected with Z^BR^ (B1, B2 and B3) were compared [Supplementary data (Fig. 9)].

*Selection analyses* - We investigated the selective regime of the polyprotein codon sites, calculating the difference (w) between the estimates of non-synonymous (dN) and synonymous (dS) substitution rates per codon site. The w values were estimated with single likelihood ancestor counting (SLAC), fast unbiased Bayes approximation (FUBAR), mixed effects model of evolution (MEME) and fixed effect likelihood (FEL) method with HyPhy program v2.11 (Pond et al. 2005), assuming a significance level (a) of 0.05. We used a maximum likelihood (ML) phylogenetic tree, inferred with GARLI v2.01 (https://code.google.com/archive/p/garli/), on polyprotein gene alignment without recombinant sequences. Codon sites under purifying selection were revealed by w < 0, and the opposite is indicative of diversifying selection. With HyPhy, we chose the best-fit substitution model (012342), based on Akaike information criterion. In addition, we calculated the Spearman coefficient of correlation between mean CAI (for Aedes and human) and posterior mean synonymous substitution rate estimated with FUBAR (the method that identified more sites under purifying selection) for each codon in ZIKV genes (data available from: https://github.com/CaioFreire/CUB).

*Phylodynamic analyses* - Using the dates of isolation, we estimated a coarse substitution rate per site per year (µ) using Path-O-Gen v1.4 (http://tree.bio.ed.ac.uk/soſtware/patho- gen/) with the ML tree for non-recombinant genomic sequences (alignment available from: https://github.com/CaioFreire/CUB). We used BEAST program v1.82 (Drummond et al. 2012), with the estimated evolutionary rate prior (m = 4.8E-4 ± 2E-4 substitution/site/year) under a relaxed lognormal molecular clock and a GTR+G+I substitution model to infer a time-scaled maximum clade credibility (MCC) tree and investigate the demographic dynamics of ZIKV lineages with the Bayesian Skyride method to estimate the temporal dynamics of effective population size (Ne.g) of ZIKV, which approximates the number of infections in time. To reveal the dynamics of viral population size growth, we calculated the Malthusian fitness (W_M_), which was approximated by the ratio of the population size in sequential time points (W_M_ = Ne.g_t_/Ne.g_t-1_).

*Ethics* - We used human iPSC, generated by cellular reprograming of Stem Cells from Human Exfoliated Deciduous Teeth (SHED), whose subjects were recruited through The Tooth Fairy Project initiative (University of São Paulo - USP), with the approval of the Ethics Committee of the Institute of Biosciences CEP-ICB/USP (Protocol CEP/ICB-USP 1001). After a complete description of this research, parents provided written informed consent for their participation.

## RESULTS

**Fig. 1:**
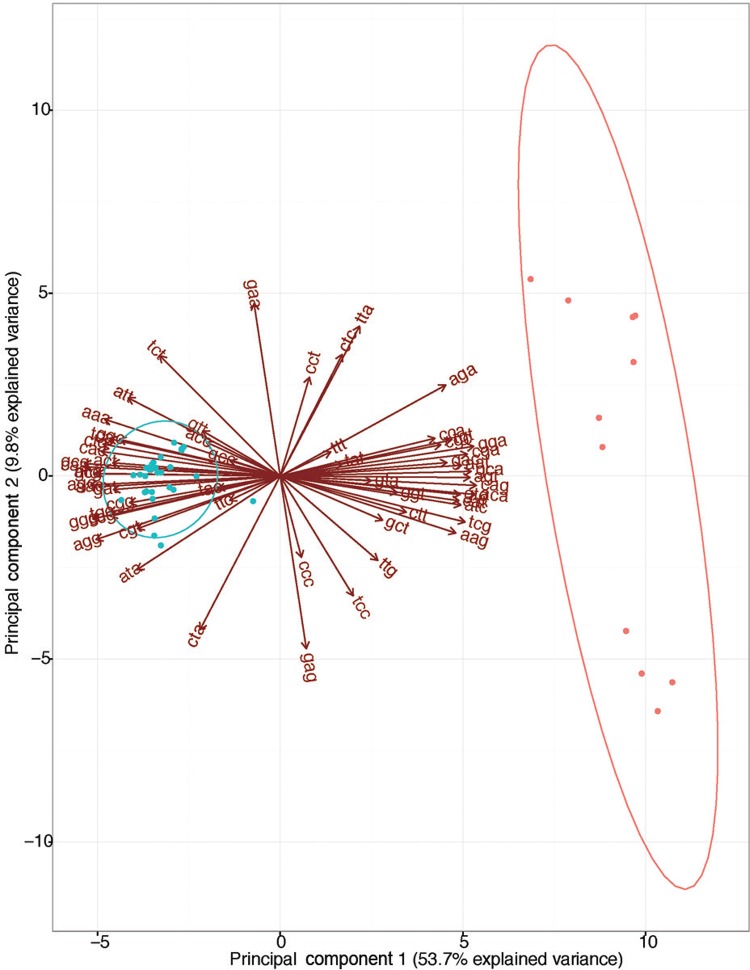
: relative synonymous codon usage analysis (RSCU) for the polyprotein coding region shows that the principal component analysis (PCA) based on RSCU agrees with the phylogenetic distinctions between the two Zika virus (ZIKV) lineages. The African (red) and Asian (blue) lineages were colour-coded accordingly. The biplot arrows indicate the preferred codons from each lineage. The ellipses delimit the groups with 95% of confidence on the biplots for PCA based on the preferential usage of codons that is lineage-specific.

**Fig. 2:**
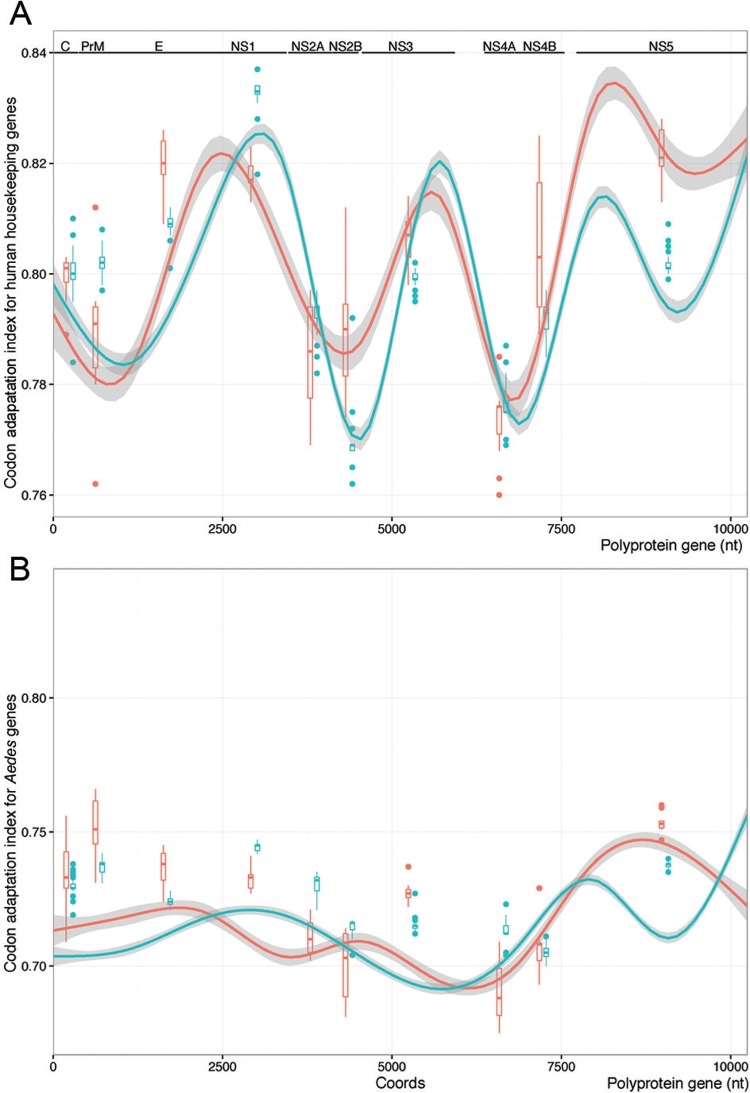
: codon adaptation index (CAI) for Zika virus (ZIKV) genes. (A) Distribution of CAI values calculated with codon usage table based on human housekeeping genes for each ZIKV gene. (B) CAI values based on *Aedes aegypti* genes. The African (red) and Asian (blue) lineages are colour-coded accordingly. The curves show sliding-window of the CAI analyses results for both, (i) the complete ZIKV polyprotein open reading frame and (ii) for each individual gene shown in colour-coded boxplots. Median, 1st and 3rd quartiles are shown in boxes and the whiskers represent the interquartile range times 1.5.

**Fig. 3:**
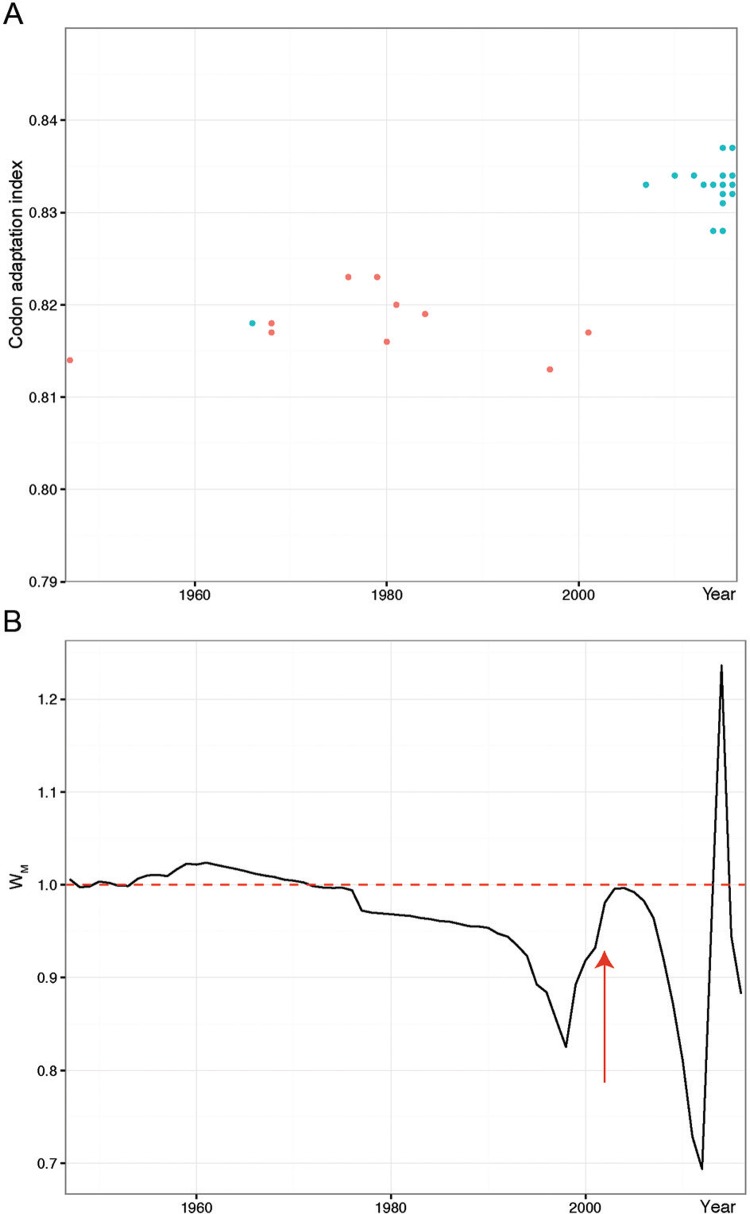
: NS1 gene codon usage and demographic dynamics of Zika virus (ZIKV). (A) NS1 gene codon adaptation index (CAI) to the human housekeeping genes for the African (red dots) and Asian (blue dots) lineages. (B) Malthusian fitness (*WM*) estimated for ZIKV since 1947, representing decrease (*wM* <1), constant population size (*wM* = 1), and net growth (*wM* > 1). The red arrow indicates the end of African lineage sampling.

**Fig. 4:**
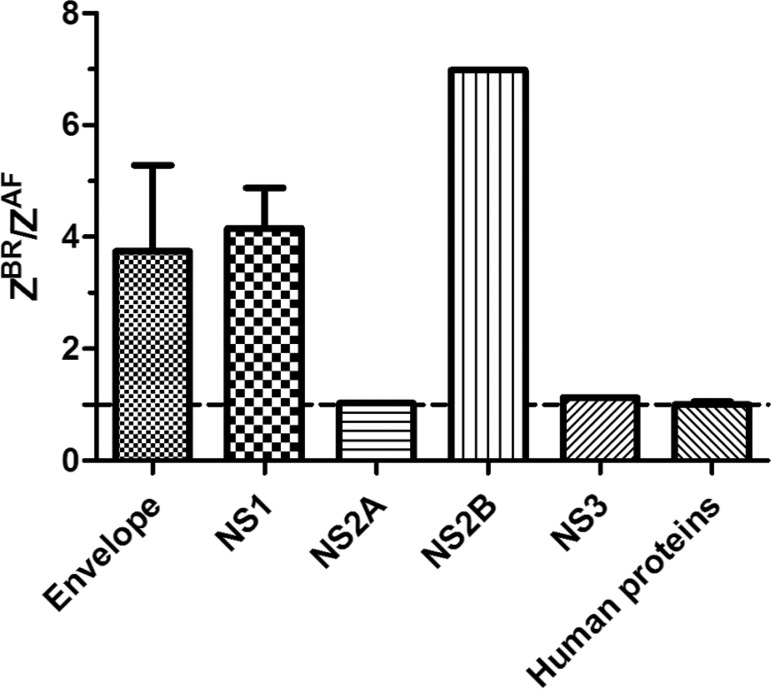
: stoichiometric ratio between proteins produced during infection experiments with the African (ZAF) and Brazilian ZIKV (ZBR) lineage analysed by mass spectrometry-based proteomics. Tryptic peptides were quantified using TMT10plex isobaric labeling and assembled into proteins. The relative expression of each viral protein from ZBR and ZAF lineages were reported as ZBR/ZAF ratio. The relative expression of 4842 human proteins identified in neurospheres infected with ZAF and ZBR was reported (data available from: https://github.com/CaioFreire/CUB). The average ratio (ZBR vs ZAF) of the human proteins is equal to 1.

*Codon preferences of ZIKV lineages are distinct* - Preferences of synonymous codons can strongly affect gene expression, which correlates with differences in infectivity. Thus, we estimated the RSCU values for each ZIKV polyprotein gene sequence (Fig. 1). By means of PCA for RSCU values, we found distinct preferences of synonymous codons in the African and Asian lineages for the entire polyprotein (Fig. 1) and for each viral gene sequences [Supplementary data (Fig. 2)]. The findings suggested an evolutionary change in codon preferences that agree with the phylogeny of ZIKV. The distinct preferences between ZIKV lineages were also observed in the analysis by absolute values of codon counts (data available from: https://github.com/CaioFreire/CUB). The extent of this codon bias was inferred by plotting the ENC that is a measure of the amount of codon preferences, ranging from 20 (extremely biased) to 61 (synonymous codons have the same probability of being used), versus the proportion of GC-content in the third position for each codon, which revealed different codon usage biases among ZIKV genes [Supplementary data (Figs 3-4)].

*ZIKV codons are under purifying selection* - We found several codon sites under purifying selective pressure (Wright 1990) [Supplementary data (Fig. 5)]. This strong purifying selection (changes on synonymous codons) was also observed for other arboviruses (Jenkins et al. 2001) and is mostly attributed to the alternation between vectors and vertebrate hosts. We also found four codons under diversifying selection, using FEL and MEME methods, which favors changes in amino acid content. Two of them were found in in the NS4B gene and the other in the NS5 (positions 2280th, 2449th, 2749th and 2807th in our alignment for the complete polyprotein gene). The MEME method revealed that 2280th and 2449th polyprotein sites were under diversifying selection 100% of the time, 2749th site was under diversifying selection 46% and 2807th was under diversifying selection in 14% of the tie. In addition, the proportion of negatively selected sites, i.e., under purifying selection, detected with the FEL method was similar among ZIKV genes (c^2^ = 7.52 and p-value = 0.58 in Pearson’s Chi-squared test with 9 degrees of freedom). Our comparisons of amino acid content showed no differences between the African and Asian ZIKV lineages (p-value = 1), obtained from the Fisher exact tests for each viral protein (C, PrM, E, NS1, NS2A, NS2B, NS3, NS4A, NS4B and NS5), in agreement with the findings that ZIKV lineages constitute a single serotype.

*Codon adaptation is different in the genes of ZIKV lineages* - Given that only the Asian lineage is associated to large human outbreaks and because we found significant distinct preferences of synonymous codons between ZIKV lineages (Fig. 1), we further compared virus codon adaptation to host and vector using *Ae. aegypti* and human housekeeping genes. We analysed the genes separately (Table, Fig. 2), since the viral polyprotein is cleaved during and post-translation (Lindenbach and Rice 2003) and the polyprotein codon usage among ZIKV strains was shown to be slightly biased (Cristina et al. 2016). We found that recent Asian epidemic lineages had a stronger codon bias in the NS1 gene (Freire et al. 2015), as indicated by its increase in codon adaptation index (CAI, blue curves in Fig. 2) (Sharp and Li 1987). Moreover, NS1 was significantly better adapted simultaneously to humans and Ae. aegypti hosts [Table, Fig. 2, Supplementary data (Fig. 7D)]. The PrM gene in the Asian lineages was well adapted to human codon usage [Fig. 2A, Supplementary data (Fig. 7)] but not for *Ae. aegypti* [Table, Fig. 2B, Supplementary data (Fig. 7B)]. On the other hand, we found that NS2A, NS2B and NS4B from the Asian lineage were significantly better adapted to *Ae. aegypti* compared to their homologues in the African lineage [Table, Fig. 2B, Supplementary data (Fig. 7)] and not to the human codon usage (Table). Importantly, in the African lineage we found that the NS4A, E and NS5 genes were significantly better adapted to humans, and the last two are also more adapted to *Ae. aegypti.* These conclusions were obtained by measurements of CAI for each ZIKV gene for both lineages, unveiling potential viral adaptation to cellular translation machinery of man and mosquitoes. Furthermore, by measuring CAI for the entire polyprotein [Supplementary data (Fig. 6)], we observed that all ZIKV strains appear to be significantly adapted to humans while they were less adapted to the *Ae. aegypti* mosquitoes (CAI values above threshold), as found for randomly sampled human genes (Cristina et al. 2016). Codons with high CAI were under strong purifying selection [Supplementary data (Fig. 8)] and we found no significant positive correlation between purifying selection and codon adaptation [Supplementary data (Table)].

**TABLE:** Codon adaptation index (CAI) for each gene of Zika virus lineages

Gene	Median CAI for human		p-value*	Median CAI for *Aedes aegypti*		p-value*
	
	African	Asian		African	Asian	
C	0.801	0.8	0.953	0.733	0.729	0.141
PrM	0.791	0.802	5.377E-5	0.751	0.738	5.499E-4
E	0.82	0.809	9.819E-6	0.738	0.724	9.423E-6
NS1	0.817	0.833	1.389E-6	0.733	0.745	8.059E-7
NS2A	0.786	0.794	0.0502	0.71	0.732	1.021E-6
NS2B	0.79	0.769	1.281E-6	0.703	0.716	1.734E-5
NS3	0.807	0.799	7.573E-4	0.727	0.715	1.892E-6
NS4A	0.776	0.775	0.8345	0.688	0.712	2.072E-6
NS4B	0.803	0.793	2.999E-3	0.708	0.705	0.4896
NS5	0.821	0.801	6.727E-7	0.753	0.738	7.563E-7

*: calculated with Wilcoxon rank sum test.

*Viral population size increase coincides with the beginning of the recent ZIKV epidemic* - We observed an abrupt increase (Fig. 3B) in Malthusian fitness (W_M_), which corresponds to variation in viral population sizes between succeeding time points, in 2013 that coincides with the breakthrough epidemic and long human-to-human chains in Pacific Islands. Crucially, all strains associated with the Asian lineage [Supplementary data (Fig. 1)] causing the recent epidemic have higher NS1 CAI values for humans than former sylvatic viruses [Fig. 3A, Supplementary data (Fig. 1)]. Our phylodynamic analyses also suggested that these codon optimisations could have happened after 1950 [Supplementary data (Fig. 1)], because CAI for the former Malaysian strain is smaller than recent Asian strains. Moreover, codon adaptation for humans in the NS1 coding region from the recent Asian lineage showed a clear increase near the present (Fig. 3A), which coincides with its spread in the Pacific and America. The strong bias in codon usage that we observed only for NS1 from epidemic strains in both hosts is suggestive of translational selection acting on this gene [Supplementary data (Fig. 7D)].

*ZIKV lineages have differences in replication and protein production* - We infected neural progenitor cells (NPC) with Brazilian (Z^BR^) and African (Z^AF^) ZIKV strains at MOI 10. Infection with both viruses presented noticeable cytopathic effect and at 24 pi yielded total RNA that was used for viral quantification by real-time polimerase chain reaction (PCR) with Cts of 24.7 (Z^BR^) and 31.7 (Z^AF^) that amounted to 1874 and 16 viral copies per ng RNA, respectively. We then compared proteins that differ from the ratios expected of the individual proteins in the viral polyprotein (Fig. 4). Our experiments indicated that Z^BR^ produced more NS1 (~4-fold), NS2B (~7-fold) and E (~3.5-fold) proteins than Z^AF^ and equal ratios of NS2A and NS3.

## DISCUSSION

*Silent selection on ZIKV* - Synonymous mutations are a common source of variation, given the constrained non-synonymous substitutions rate imposed to RNA viruses that have to negotiate successful infections, alternating between humans and mosquitoes. Assuming codons as evolutionary units of selection, we could explain CAI heterogeneity along the genome due to silent selection, in a similar fashion as the fixation of specific amino acid replacements due to drift or positive selection, once they confer selective advantage to the organism (Brandis and Hughes 2016). Nevertheless, during cell line infection, a single polypetide is expected to be produced from the complete ORF encoded by the viral genomic RNA and this is assumed as the canonical mechanism of translation in the genus Flavivirus. Therefore, there is no clear explanation for how selection would act preferentially at some codons of specific ZIKV genes that are believed to be co-replicated and are believed to be co-translated. Given the observed differences in CAI and the stoichiometric differences found among viral proteins, other aspects could to be considered. For example, the complex biology of these viruses that infect several tissues of insects and vertebrates, several mechanisms could account for translational selection being applied differently at different portions of the polyprotein. These could include both replicative and translational processivity differences, for example. In this regard, the genomic organisation of flaviviruses has the structural proteins, which are usually necessary in larger amounts for viral morphogenesis, positioned in a way that they are translated before the nonstructural proteins, mostly enzymes necessary in lesser amounts than the structural proteins.

It is noteworthy that NS1 is also necessary in large amounts and is located at the border between structural and non-structural genes. Interestingly, an internal translation initiation was postulated for Flavivirus RNA downstream form the NS1 (Westaway 1977), possibly at a secondary ribosomal attachment site, on the basis of ultraviolet inactivation experiments with the Kunjin virus (Westaway et al. 1984). A pseudoknot at the coding region for the C-terminal of the WNV NS1 is associated with a -1 ribosomal frame shift leading to the translation of a longer form of NS1 (Brinton 2013). Moreover, non-canonical, 5’ cap-independent translation initiation has been shown for DENV (Edgil et al. 2006) and initiation at non 5’ proximal AUG triplets is a common feature of two other genera (Hepacivirus and Pestivirus) of the family Flaviviridae. These features could account for decoupling selective forces among genes of flaviruses in general and ZIKV in particular.

*Biological correlates of codon usage in ZIKV* - The higher values of CAI and the higher viral loads produced by Z^BR^ compared to Z^AF^ in NPC cells did agree with the ratios observed in our proteomics data for NS1 (Fig. 4). Accordingly, we detected more viral proteins in NPC infected by Z^BR^ than by Z^AF^. However, these differences were not exclusively explained by the differences in titration between the viral lineages. We detected equal proportions of NS2A and NS3 proteins, but a relative increase in NS1, NS2B and E proteins in Z^BR^ infected cells (Fig. 4). The differences in ratio could also be explained by stability differences in the proteins being produced in the two viral lineages. Nevertheless, the agreement of CAI values and stoichiometric ratios could also entail translational efficiency differences. It has been shown that codon usage composition correlates with observed variability in protein:transcript ratios. Moreover, viral codon usage optimisation was also shown to be associated with fine-tuning viral host interactions (Longdon et al. 2014) and the most affected viral genes are usually those highly expressed (Bahir et al. 2009). It is noteworthy that the NS1 protein is secreted by infected cells as hexamers that are implicated in immune evasion strategies by blocking complement-mediated responses during infection in humans and, as a dimers, plays an important role in viral replication (Müller and Young 2013), suggesting a possible advantage for NS1 increased production.

Crucially, codon adaptation is more critical for human viruses than for viruses that infect other mammals (Bahir et al. 2009), which could be the case of the recent epidemic Asian ZIKV lineage in contrast to the African lineage. Although our experimental data agrees with our results on different CAI values for NS1, the differences in the stoichiometry of ZIKV proteins we observed indicate that additional factors, *e.g.*, differences in protein stability could be implicated. The lower CAI for *Ae. aegypti* that we observed for all ZIKV genes could be associated to the use of *Ae. aegypti* as ZIKV vector in South and Central America (Chouin-Carneiro et al. 2016), while several other *Aedes* spp. mosquitoes are its vectors in Africa and ZIKV transmission by *Ae. aegypti* from Africa is insignificant (Diagne et al. 2015). Moreover, *Ae. aegypti* from the Americas was shown to have low competence for ZIKV in the laboratory (Chouin-Carneiro et al. 2016). The elevated CAI for humans compared to mosquitoes has also to be considered under the perspective of a possible role of persistence in humans, since ZIKV was shown to be present and transmissible in semen (Barzon et al. 2016), and other modes of transmission.

Moreover, the recent spread of ZIKV to French Polynesia and the Americas has been associated to unusual increase in congenital malformation (*e.g*., microcephaly) and GBS reporting (Ioos et al. 2014). Although we show: (i) some evidence of changes in codon usage in Asian lineage of ZIKV, which were independently confirmed (Cristina et al. 2016), (ii) our additional proteomics findings on stoichiometric differences between these lineages and (iii) given that they constitute a single serotype; it remains to be shown if CZS is a specific outcome of the infection by the Asian lineage in the Americas.
