# Supplementary material for: NS1 codon usage adaptation to humans in pandemic Zika virus
Source: Mem Inst Oswaldo Cruz. 2018 May 10;113(5):e170385. doi: 10.1590/0074-02760170385 (PMC5942634; doi:10.1590/0074-02760170385)
Supplement: Supplementary file 1 [file 0074-0276-mioc-113-5-e170385-Suppl01.pdf]

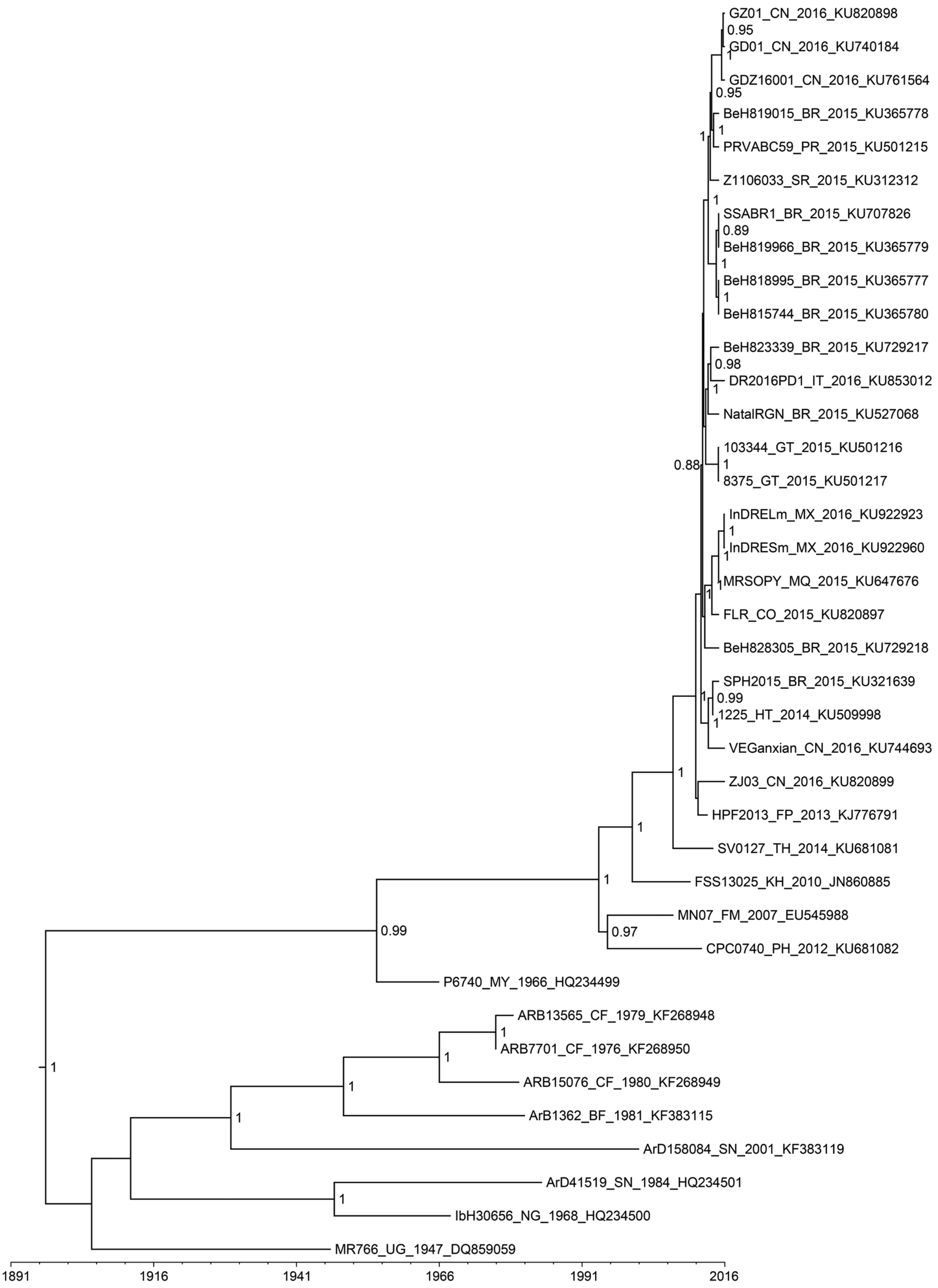

Fig. 1: time-scaled maximum clade credibility tree for non-recombinant Zika virus (ZIKV) complete coding genomic sequences. The posterior probability values are shown close to the nodes on tree and the temporal scale is shown in the bottom of the figure.

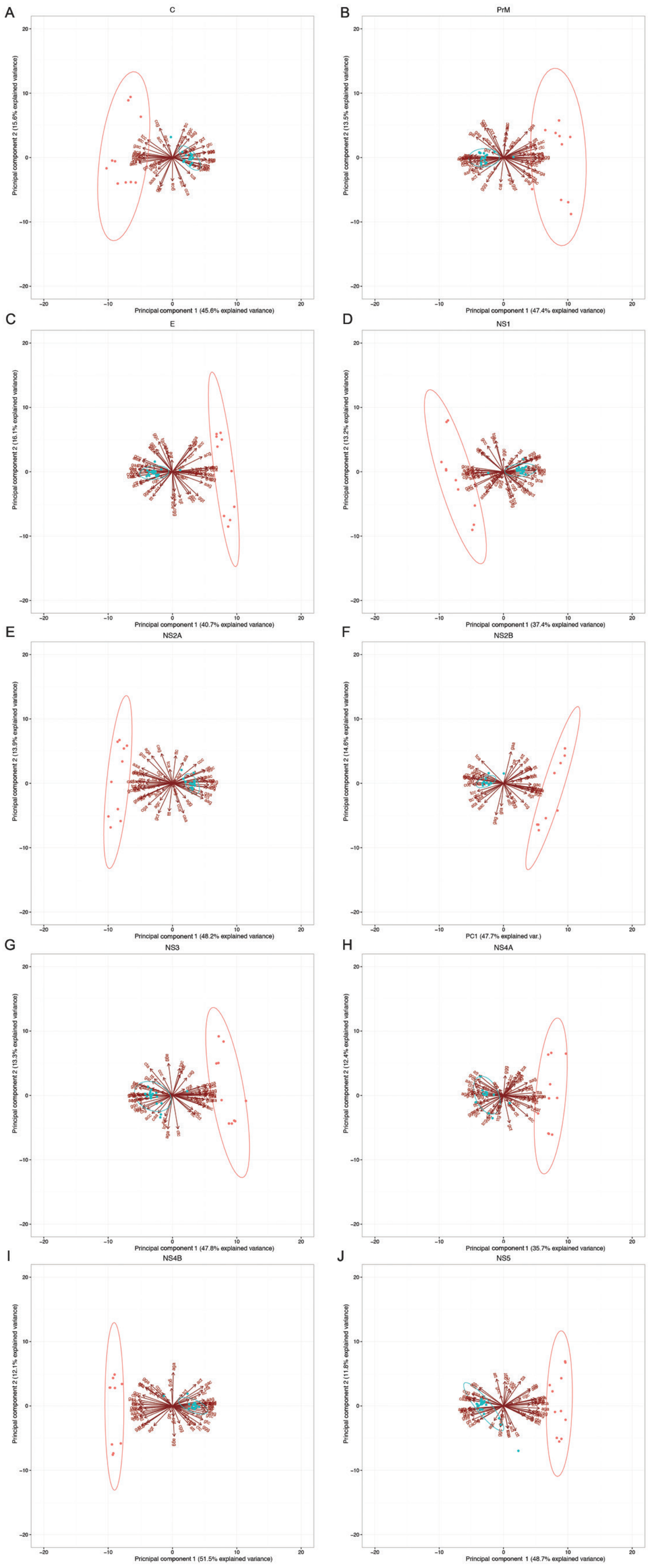

Fig. 2: codon preferences by principal component analyses on relative synonymous codon usage (RSCU) for each Zika virus (ZIKV) gene. Strains from African lineage are shown as small red circles and the Asians as blue circles. The biplot arrows indicate the preferred codons from each lineage. The ellipses delimit the groups with 95% of confidence on the biplots for principal component analysis (PCA) based on the preferential usage of codons that is lineage-specific. (A) Biplot for Capsid gene. (B) PrM. (C) Envelope. (D) NS1. (E) NS2A. (F) NS2B. (G) NS3. (H) NS4A. (I) NS4B. (J) NS5.

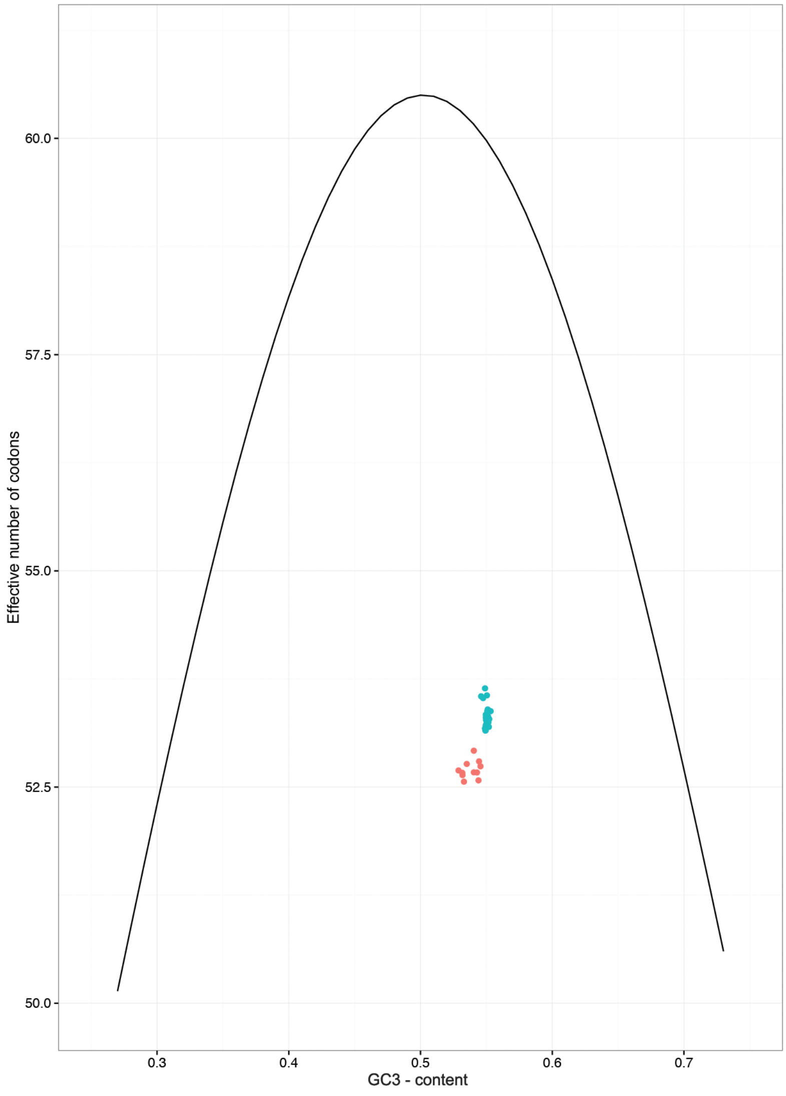

Fig. 3: effective number of codons (ENC) against GC-content on the third base for the polyprotein gene of Zika virus (ZIKV) lineages. The Asian lineage was colour-coded in blue and African in red. The solid curve represents the expected ENC when codon usage is only determined by GC3-content.

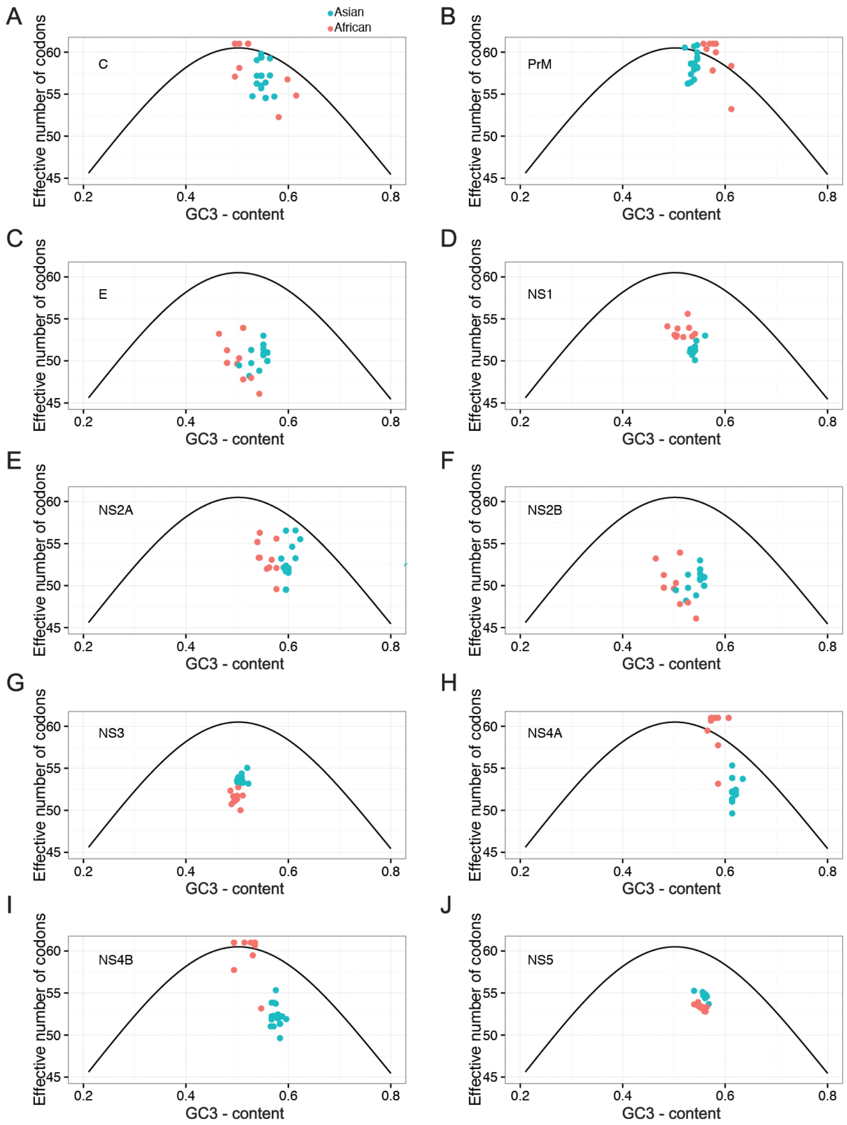

Fig. 4: effective number of codons (ENC) against GC-content on the third base for each gene of Zika virus (ZIKV) lineages. The Asian lineage was colour-coded in blue and African in red. The solid curve represents the expected ENC when codon usage is only determined by GC3-content. (A) Capsid gene. (B) PrM. (C) Envelope. (D) NS1. (E) NS2A. (F) NS2B. (G) NS3. (H) NS4A. (I) NS4B. (J) NS5.

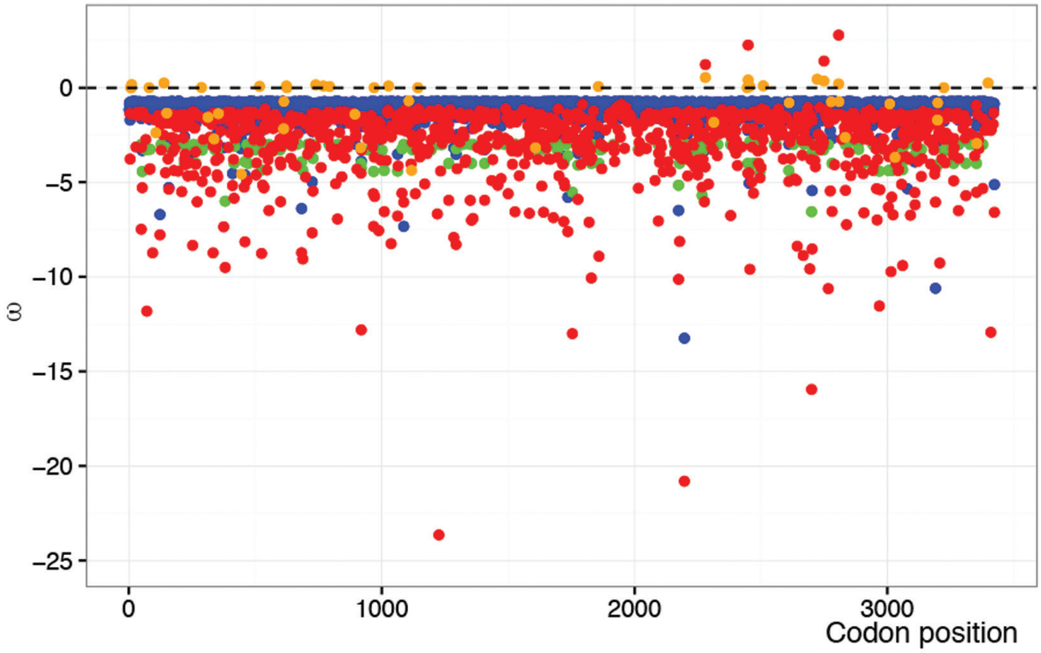

Fig. 5: codon sites under significant selection detected for the polyprotein gene of Zika virus (ZIKV). The detected codons were colour-coded according to the employed method: blue for fast unbiased Bayes approximation (FUBAR); green, single likelihood ancestor counting (SLAC); red, fixed effect likelihood (FEL) and orange, mixed effects model of evolution (MEME). Codon sites under purifying selection were revealed by w < 0, and the opposite is indicative of diversifying selection. The dashed line marks the neutral selection (w = 0). To improve the visualisation, we omitted two points of this graph: (i) SLAC result for codon 170, w < -60.20 and (ii) FEL, for 2975 w = -407.48.

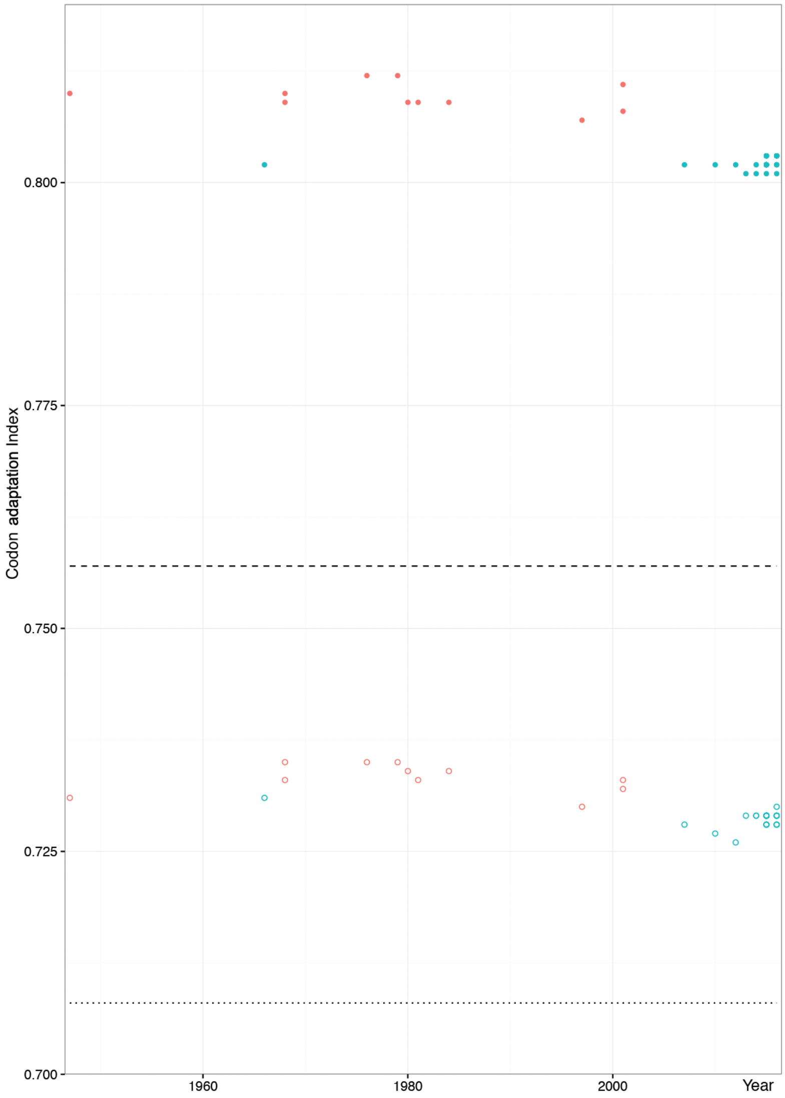

Fig. 6: codon adaptation index (CAI) for polyprotein gene of Zika virus (ZIKV) lineages according to year of isolation. Solid circles represent the CAI values obtained using a codon usage table based on human housekeeping genes and dashed line marks the threshold of significance. Empty circles represent the CAI values obtained with *Aedes aegypti* codon table and the dotted line marks their threshold.

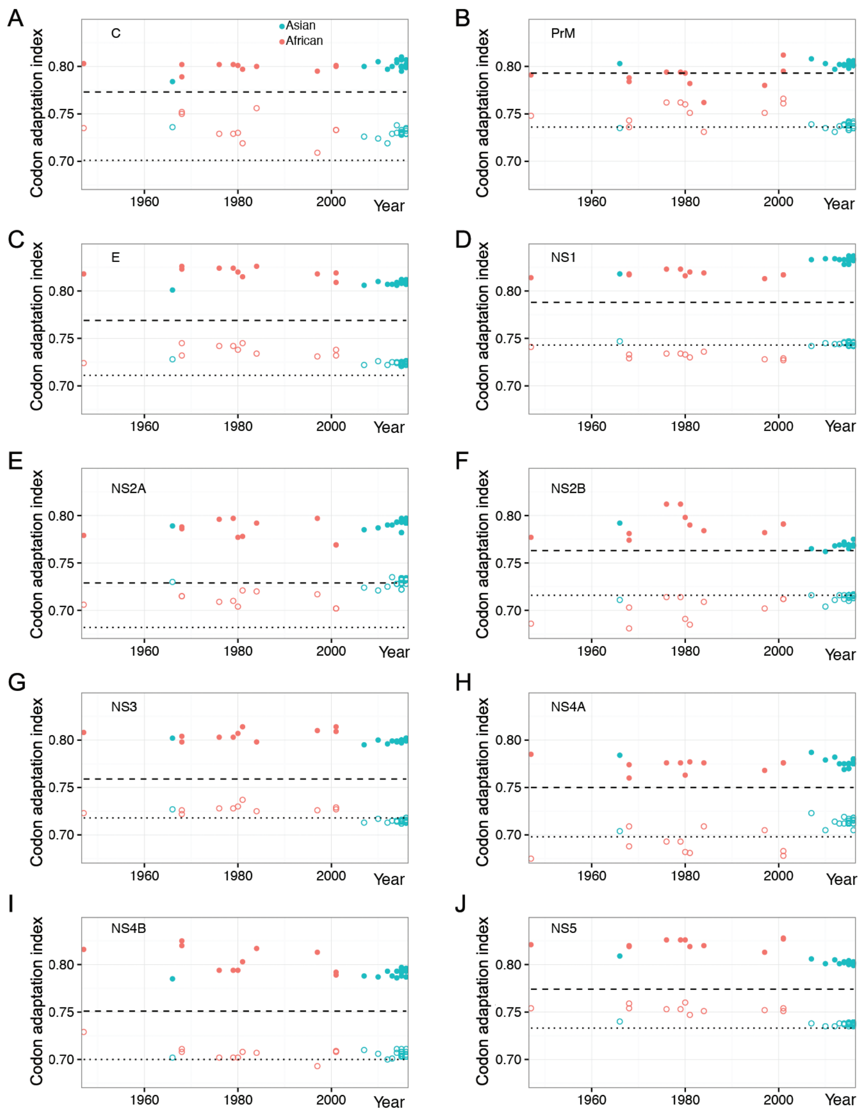

Fig. 7: codon adaptation index (CAI) for each gene of Zika virus (ZIKV) lineages according to year of isolation. Solid circles represent the CAI values obtained using a codon usage table based on human housekeeping genes and dashed line marks the threshold of significance. Empty circles represent the CAI values obtained with *Aedes aegypti* codon table and the dotted line marks their threshold. (A) CAI values for Capsid gene. (B) PrM. (C) Envelope. (D) NS1. (E) NS2A. (F) NS2B. (G) NS3. (H) NS4A. (I) NS4B. (J) NS5.

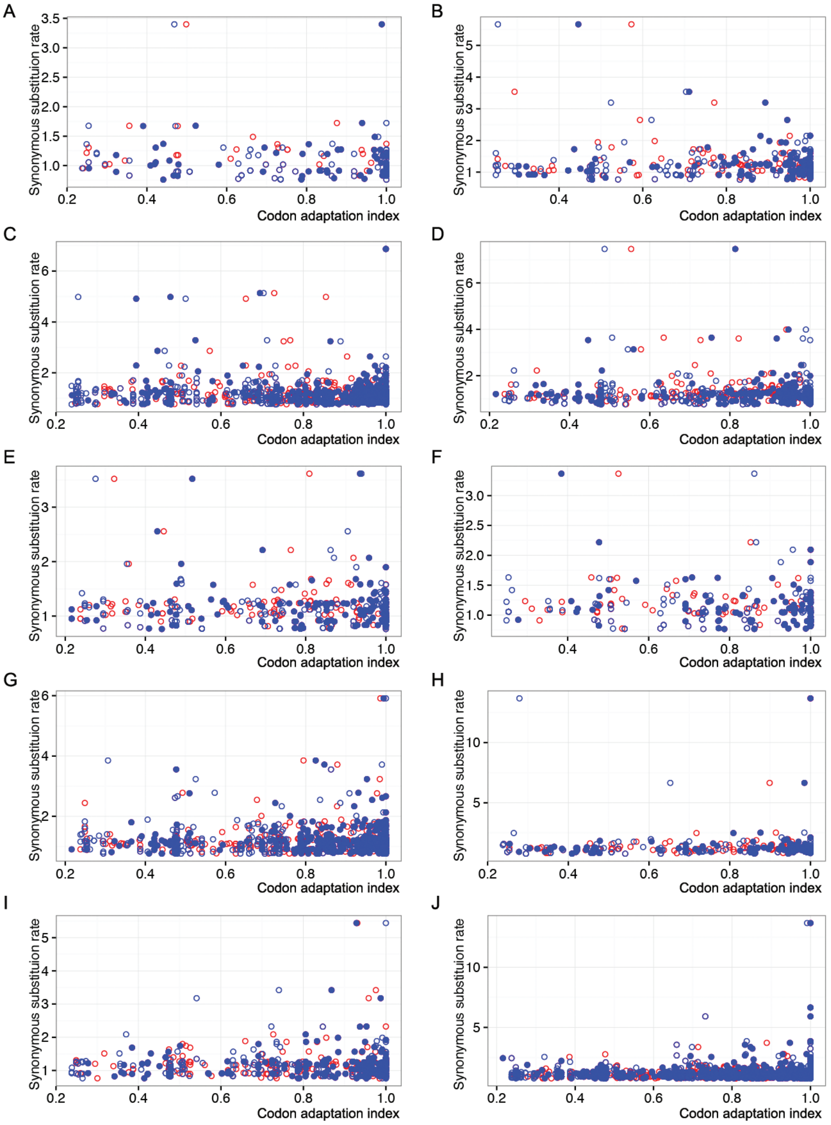

Fig. 8: correlations between codon adaptation index (CAI) values and synonymous substitution rates for each codon of Zika virus (ZIKV) genes. The empty circles represent mean CAI values for *Aedes aegypti* and the solid ones the mean CAI values for humans. The ZIKV lineages were colour-coded with blue to Asian and red for African. (A) Capsid gene. (B) PrM. (C) Envelope. (D) NS1. (E) NS2A. (F) NS2B. (G) NS3. (H) NS4A. (I) NS4B. (J) NS5. The CAI values for Methionine and Tryptophan were not shown since they are coded by a single codon: ATG or TTG, respectively.

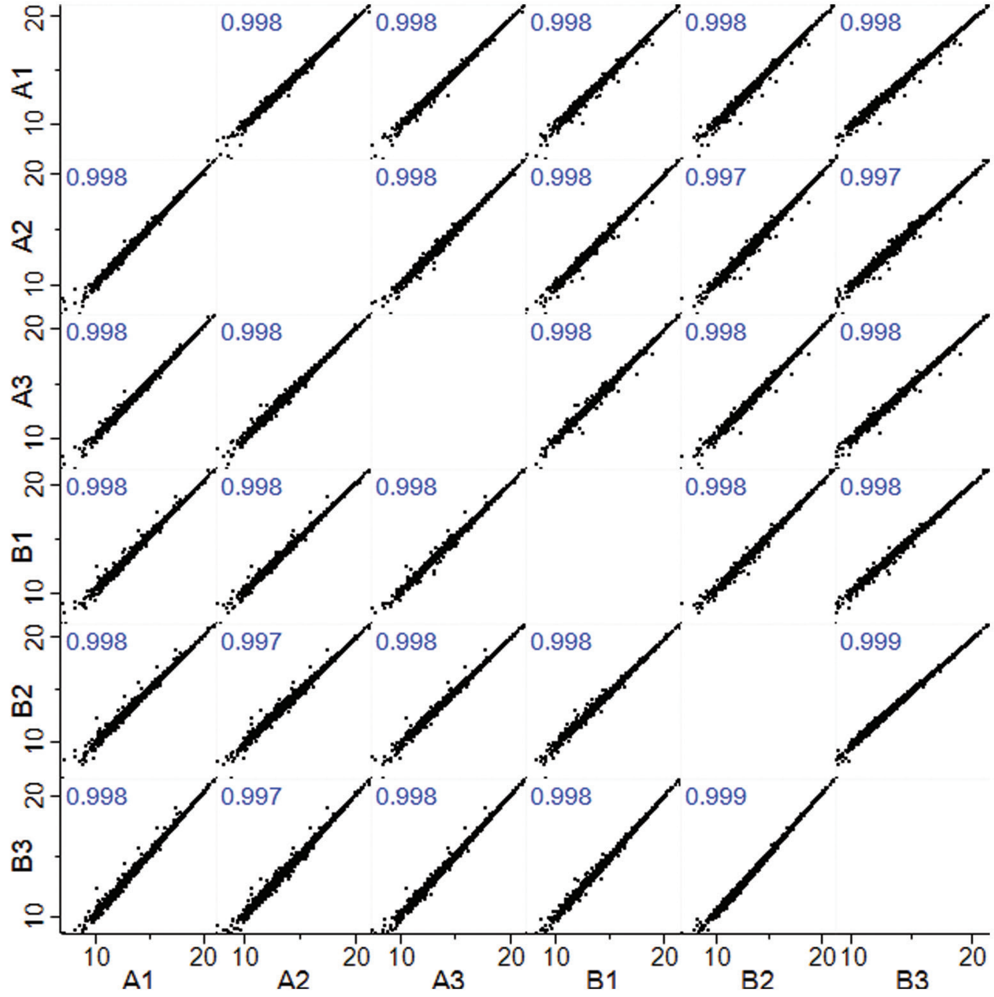

Fig. 9: evaluation of biological variability of neurospheres infected with Zika virus (ZIKV). The log2(Intensity) for each protein identified and quantified was compared and reported as a scatter plot. The three biological replicates of neurospheres infected with Z^{AF} (A1, A2 and A3) and three biological replicates of neurospheres infected with Z^{BR} (B1,B2 and B3) were compared.

TABLE

Spearman's rank correlation coefficient calculated with codon adaptation index (CAI) values and synonymous substitution rates for each codon of Zika virus (ZIKV) genes

	CAI from African lineage for human genes		CAI from African lineage for *Aedes aegypti* genes		CAI from Asian lineage for human genes		CAI from Asian lineage for *Ae. aegypti* genes	
Gene	r*	p-value	r	p-value	r	p-value	r	p-value
C	-0.1786389	0.09984	-0.2719056	0.01132	-0.1538632	0.1572	-0.2676998	0.01271
PrM	-0.1306902	0.1074	-0.1910138	0.01802	-0.07744972	0.3413	-0.160921	0.04691
E	-0.165376	0.0004026	-0.1108614	0.01813	-0.1826981	9.04E-05	-0.1311563	0.005126
NS1	-0.2012694	0.0003038	-0.1125193	0.04497	-0.1474766	0.00844	-0.1130614	0.04394
NS2A	-0.1894806	0.008836	-0.1934817	0.00748	-0.1870377	0.009767	-0.2188672	0.002415
NS2B	-0.2186324	0.0189	-0.2156239	0.02065	-0.2732838	0.003126	-0.203058	0.02952
NS3	-0.1233101	0.01124	-0.05388879	0.2694	-0.1273739	0.008806	-0.1147934	0.01833
NS4A	-0.163526	0.05714	-0.1233215	0.1526	-0.1542678	0.07294	-0.140591	0.1026
NS4B	-0.2264145	0.0006771	-0.2280998	0.0006155	-0.1690593	0.01164	-0.2354767	0.0004022
NS5	-0.06170454	0.09828	0.01876869	0.6012	-0.0140327	0.6959	0.0121993	0.7341

*: Spearman's rank correlation coefficient.
